# Health and environment from adaptation to adaptivity: a situated relational account

**DOI:** 10.1007/s40656-022-00515-w

**Published:** 2022-08-18

**Authors:** Laura Menatti, Leonardo Bich, Cristian Saborido

**Affiliations:** 1 Department of Philosophy, IAS-Research Centre for Life, Mind and Society, University of the Basque Country (UPV/EHU), Avenida de Tolosa 70, 20018 Donostia-San Sebastian, Spain; 2 Center for Philosophy of Science, University of Pittsburgh, 1117 Cathedral of Learning, 4200 Fifth Ave., Pittsburgh, PA 15213 USA; 3 Department of Logic, History and Philosophy of Science, UNED, Paseo de la Senda del Rey 7, 28040 Madrid, Spain

**Keywords:** Health, Medicine, Environment, Adaptation, Adaptivity, Philosophy of medicine, Environmental philosophy, Philosophy of biology

## Abstract

The definitions and conceptualizations of health, and the management of healthcare have been challenged by the current global scenarios (e.g., new diseases, new geographical distribution of diseases, effects of climate change on health, etc.) and by the ongoing scholarship in humanities and science. In this paper we question the mainstream definition of health adopted by the WHO—‘a state of complete physical, mental and social well-being and not merely the absence of disease or infirmity’ (WHO in Preamble to the constitution of the World Health Organization as adopted by the international health conference, The World Health Organization, 1948)—and its role in providing tools to understand what health is in the contemporary context. More specifically, we argue that this context requires to take into account the role of the environment both in medical theory and in the healthcare practice. To do so, we analyse WHO documents dated 1984 and 1986 which define health as ‘coping with the environment’. We develop the idea of ‘coping with the environment’, by focusing on two cardinal concepts: adaptation in public health and adaptivity in philosophy of biology. We argue that the notions of adaptation and adaptivity can be of major benefit for the characterization of health, and have practical implications. We explore some of these implications by discussing two recent case studies of adaptivity in public health, which can be valuable to further develop adaptive strategies in the current pandemic scenario: community-centred care and microbiologically healthier buildings.

## Introduction

Health is a debated issue in philosophy of science and philosophy of medicine, together with the concepts of disease, pain and well-being, just to mention a few.^1^ Recent events, such as the COVID-19 pandemic, the diffusion of other diseases beyond their original geographical boundaries, and the effects of climate change on health, have radically challenged the management of healthcare and health policy, by showing the need to discuss and to widen the concept of health.

Our contention is that to understand and to promote health in the current scenarios, a new situated and relational account needs to be introduced, capable to take into account the relationship between health and the environment.

Historically, the environment has received little attention in the definitions of health and disease proposed by medical organizations. For example, in the preamble to its Constitution, the World Health Organization (WHO) defines health as “a state of complete physical, mental and social well-being and not merely the absence of disease or infirmity” (WHO, 1948). The concept of the environment does not appear in this definition, which, even if introduced in the historical context of the years immediately following World War II, nonetheless continues to be the most used in medical sciences and medical education (for a history of this definition see Valles, 2018, p. 33).

More recently, a long list of contemporary international documents—such as the Millennium Ecosystem Assessment (see Reid et al., 2005) or the *OneHealth* approach (see Mackenzie & Jeggo, 2019; see also the concept of planetary health in Horton et al., 2014)—have been calling for the importance of the environment in healthcare and medical theory. However, a specific relational conceptualization of health as related to the ‘environment’ has not been provided. A mention of the environment, for instance, appears in the long list of Social Determinants of Health (SDH, first introduced in 2008; Commission on Social Determinants of Health, 2008; see also Committee on Educating Health Professionals to Address the Social Determinants of Health, 2016; see also Solar & Irwin, 2010), when it refers to “housing, basic amenities and the environment”, together with the various conditions which have an impact on health, among them: governance, education, employment, social security, etc. The SDH approach is an important tool for studies relating sociology with health. In medical practice, the SDH are valuable in determining health risks and preventing disease in a given population (e.g., Cockerham et al., 2017; Scribner et al., 2017). In health management, SDH have informed political initiatives such as ‘healthy people’ in the United States.^2^ SDH also play an important role in the recent theoretical and practical account of population health (Valles, 2018). However, the reference to “the environment” in the SDH is generic. The environment is reduced to an item in a list and its role is equated to other numerous variables in the life of human beings.

Turning to philosophy of medicine, the environment is often implicit or controversially characterized. In the debate about the definition of health and disease, a distinction is often made between two main currents: *naturalism* and *normativism* (for a review of this debate, see Kovàcs, 1998; Khushf, 2007; Giroux, 2016). The role of the environment is different for each of these approaches.

Naturalists (also called “biologicists”, “mechanicists”, and “reductionists”) seek a notion of health—as well as of its reverse, disease– in value-free terms (Boorse, 1977, 1997; Lennox, 1995; Scadding, 1990). According to naturalism, a definition of health and disease must be able to offer objective criteria applicable to all individuals of the same type. The most influential naturalistic approach, Christopher Boorse’s “biostatistical approach” (1977; 1997), identifies health with the typical biological functioning of the majority of members in a reference class, which Boorse considers to be individuals of the same species, sex and age. Consequently, ‘health’ would be not something dependent on the particular environment in which individuals live, but the biologically normal functioning of an organism, and this normal functioning can only be specified with respect to a standardised environment, which naturalists have called “normal” (Boorse, 1977, 1997), “relevant” (Hausman, 2011), or “appropriate” (Garson & Piccinini, 2014).^3^

However, the use of the notion of ‘normal environment’ to account for health and disease has been considered as problematic. Elselijn Kingma (2010) has pointed out that the biostatistical conception of biologically correct functioning behind naturalistic approaches to health must be relativised to organismal and environmental situations or ultimately rejected. The same functioning can be correct in relation to one situation and incorrect in relation to another. For example, Kingma argues that in the case of a paracetamol overdose, liver function is extremely low. This low functioning may intuitively be understood as dysfunctional, but it is nevertheless statistically typical for the situation of paracetamol overdose. One could argue that paracetamol overdose is not a typical situation for the biological species—the biological design has not been evolutionarily shaped in such situations—but this problem of situation-specificity can also be seen in much more typical cases such as malaria. Organic design has indeed been evolutionarily shaped in environments with *Plasmodium falciforum*, as well as with many other pathogens, and these pathogens are therefore part of the ‘normal’ environment for naturalists. In sum, the naturalistic approach relies on a standardised conception of the environment, characterized as a set of background or boundary conditions. This makes it difficult to understand the role played by environmental factors and, more generally, the complexity of the relationship between health and environment.

In contrast, the constructivist or normativist view is based on an entirely different assumption, as it explicitly recognises the situation-specificity of notions of health and disease. For normativist theorists (Margolis, 1976; Engelhardt, 1986; Nordenfelt, 1987; Fulford, 1989) notions of health and disease are inevitably value-laden and it is not desirable, or even possible, to achieve an objectivist definition of healthy and diseased states. The states that can be considered as healthy or diseased correspond to ways of functioning that are positively or negatively evaluated (at least partially) on the basis of subjective criteria. Notions such as ‘suffering’, ‘impairment’ or ‘well-being’^4^ are central to this interpretation of health and disease, and are notions laden with subjective values. In this line, for example, Lennart Nordenfelt (1987, 2007) defines health as the “ability to reach the subject’s vital goals”, and these vital goals are “preconditions for the subject’s minimal happiness” (Nordenfelt, 2016, p. 222). Assuming that what defines health is a subjective evaluation—in this case, what is a of the “minimum happiness” for each individual– implies that the evaluation of the relationship with the environment for health is also subjective. This approach therefore entails a “contextualism” in which the particularities of a specific individual-environment relationship must be observed in order to determine health and disease (see Rezneck, 1987; Sholl, 2016).

However, authors such as Boorse (1977) have argued that this type of contextualism leads to a problematic relativism. Understanding health in terms of individuals’ ‘success’ or ‘efficiency’ in relation to their environment, implies that the same state could be categorised as either healthy or pathological depending on subjective criteria (e.g., myopia or obesity, could be a disease for some and a completely healthy state for others). For normativists, the efficiency of a state is something that will depend on individuals’ evaluation of their environment, identifying opportunities and limitations. Yet, as Woolfolk (1999) has pointed out, contextualism implies a relativistic approach that does not allow us to identify misuses of medical notions (e.g. diagnosing and treating political dissidents, homosexuals or left-handed people as diseased people) and falls into a problem of under-specification of health and disease.

Interestingly, when the environment is not overlooked, reduced to a standardised set of boundary conditions, or loosely characterised in terms of context, it is often characterized as pathogenic. As an example, let us think of these years of pandemic: the environment has been generally characterized as a risk to human health and a source of pathogenic elements: e.g., a carrier of diseases, toxic substances, etc. The idea of the environment as the carrier of viruses has indeed spread since January 2020. Along with a stigmatizing narrative about China, its inhabitants and cultural traditions (see Xu et al., 2021), the environment—more specifically animals—has often been reduced to terms such as spill-over and zoonosis, related to the hunting or commercialization of the animal (bat or pangolin) thought as responsible for the zoonosis of COVID-19 (Santana, 2020). We acknowledge that these concepts have positively conveyed the popularization of scientific language, of the modes of transmission of different viruses, and the beginning of an awareness of the larger role of viruses in the biosphere, in human physiology and evolution (see Quammen, 2012/2020; Zimmer, 2020). However, we think that the prevalent idea communicated and discussed is that of the environment as pathogenic. This idea—consubstantial to medicine and medical education—has increased the polarization between human beings and their health on one side, and the environment on the other.^5^ This is detrimental to the understanding of the negative as well the positive elements of the environment.

By considering these two premises—the forgotten role of the environment and its opposition to health due to its exclusively pathogenic characterization—in this paper we take a first step towards developing an understanding of the relationship between health and environment, leading to a situated relational perspective. Our aim is twofold, theoretical and pragmatic: to develop a new conceptual characterization of health that allows to think and discuss the role of environment and has operational outcomes for the management of healthcare, medical education, and the design of buildings and outdoor public spaces (built and natural).

To do so, in Sect. 2 we develop a brief history of the relationship between health and the environment in the medical sciences. We show how the discussion of the environment has tended to be progressively abandoned after the emergence of germ theory. In Sect. 3 we analyse documents published by WHO in 1984 and 1894 which innovatively define health as “coping with the environment” and “creating supportive environments” but, significatively, are not widely known and employed. By building on the insights provided in the 1984 and 1986 documents, we develop the idea of health as ‘coping with the environment’ by focusing on the concepts of adaptation from public health (Sect. 4), and of adaptivity from philosophy of biology and theoretical biology (Sect. 5). We argue that the philosophical notion of adaptivity allows to achieve a better understanding of the role of the environment with respect to health. In doing so, we also rethink the environment not only as a set of external boundary conditions, or as a source of perturbations, but in relational terms as interacting with organisms and as itself part of those processes aimed at actively promoting the conditions of existence of living organisms (Sect. 6). We illustrate the pragmatic implications of these ideas by discussing two recent case studies in terms of adaptivity as creation of supportive environments applied to the health care management: community-based care and bioinformed microbiologically healthier buildings (Sect. 7).

## A brief history of the relationship between health and environment

The conceptual relationship between environment and health can be traced back to the origins of medicine. Starting from the *Hippocratic corpus* (V BCE—II CE) the role of the environment has been causally related to several symptoms and conditions. In the book *On Airs, Waters and Places* of the *Hippocratic corpus,* geography and meteorology play an important role in determining human traits, and are related to conditions such as diarrhoea, malaria, and catarrh (Berridge et al., 2012; Karamanou et al., 2012; Porman, 2019; Porter, 1998). Humoral pathology, based on the balance of the four humours (blood, phlegm, yellow bile, and black bile) derived from the *Corpus Hippocraticum* and Galen’s theory, remained fundamental in medical epistemology and practice all over the Renaissance and up to the nineteenth century (Porman, 2019). Humoral pathology was often accompanied by miasma theory, which was predominant until the introduction of germ theory and is no longer accepted by the scientific community. Miasma in ancient Greek means stain, contamination, and pollution. Miasma was characterized as a poisonous and smelly vapour filled with rotten particles/elements and it was considered responsible for disease. Miasma theory states that diseases (such as cholera) come from a miasmatic environment. The influence of miasma theory can still be found in the etymology of many diseases. An example is the term *malaria,* introduced in medieval Italian from the contraction of the Italian words *mala aira* (bad air); similarly, the Latin term for this disease is *paludism* (disease of the marshes) (Porter, 1998, p. 20). In the context of miasma theory, the environment was mostly identified with air.

Miasma theory was famously questioned by John Snow during the epidemic of cholera in London in the middle of the nineteenth century and later, at the end of the nineteenth century, it was replaced by germ theory by Pasteur and Koch (Bingham et al., 2004; Gaynes, 2019; Gradmann, 2009; Porter, 1998). The introduction of germ theory radically changed the way the relationship between environment and health was understood. According to some analysis, the scientific revolution of germ theory may have intensified the separation between health and environment by focusing more on the individual than on the environment (Berridge et al., 2012, p. 1). Germ theory is more narrowed in the identification of the causative germ of a disease, in isolating it and targeting it with countermeasures, rather than considering a wider range of environmental factors. The framework of germ theory establishes a clear distinction between the organism and the environment: it is the organism that, in contact with an external agent, can become ill. The environment is considered as a source of potentially pathogenic elements. However, medical practice and preventive medicine had carried out a negotiation between environmental hygiene and germ theory, not only in tropical medicine but also in town planning and urban design (Schott, 2012, p. 71; Casanova & Abel, 2013).

During the history of medicine, the pathogenic account of the environment remains preponderant as the biomedical efforts are concentrated on individuating the causal germ responsible for a specific disease. We argue that the salutogenic (*salus* = health/*genic* = originating, producing) account of medicine should complement the pathogenic ones. Salutogenesis—which has been firstly developed by Antonovsky (1979)—basically refers to the fact that health sciences need to be focused on the plurality of causes of health and disease, on prevention and on health promotion, by taking into account the ongoing environmental and social life courses of the individuals and social groups (see also Mittlemark et al., 2017). The salutogenic approach has influenced public health, population health and documents such as the WHO Ottawa Charter or the Leeds Declaration (see Valles, 2018, p. 119). Moreover, it is important to mention that the positive and the preventive effects of the environment have been implemented, starting from Hippocrates until the contemporaneity (e.g., preventive medicine, dietary restrictions and suggestions, etc.). In addition, contemporary psychology has been focusing on restorative or salutogenic environments. Research and evidence-based studies in psychology and in biomedical sciences analyse the beneficial effects of greenery and green spaces on health and well-being of patients, healthcare professionals and various categories of individuals. In this sense the environment has been characterized in terms of natural and human-made surroundings, by employing concepts such as landscape, green spaces, greenery as used in contemporary psychology and philosophy, landscape architecture and design (Menatti & Casado, 2016). The idea of ‘supportive environments’ that we will develop in the following sections is in line with and complements this research.

During the past decades, the role of the environment has reappeared mostly in the form of climate change issues: “the reformulations of public health and new environmentalism went hand in hand” (Berridge et al., 2012, p. 1). For instance, in 2009, the journal *The Lancet*, together with the Institute for Global Health Commission of the University College of London (UCL) published a special issue: *Managing the health effects of climate change* (Costello et al., 2009). According to Berridge et al. (2012), this publication is a watershed in the reconsideration of the health–environment relationship. However, the role of the environment is restricted to the effects of one specific phenomenon occurring on a global scale: climate change. Specifically, medical doctors, together with the UCL Commission, underline the decrease in biodiversity and climate change as major problems in global health. Climate change effects, it is argued, will exacerbate the differences between rich and poor and they will have enormous repercussions on health and access to health services for everyone. The adaptation to climate change is analysed with respect to: “changing patterns of disease and mortality, food, water and sanitation, shelter and human settlements, extreme events, and population and migration. Many health problems—such as malnutrition, diarrhoea, cardiorespiratory and infectious diseases—need to be considered as effects of global average temperature change” (Costello et al., 2009: Fig. 1, p. 1700). A new public health movement is advocated in order to reduce and treat the effects of climate change on the health of global populations.

Currently, a long list of international official documents—such as the Millennium Ecosystem Assessment (see Reid et al., 2005) or the *OneHealth* approach (see Mackenzie & Jeggo, 2019)—call for recognition of the importance of the environment in healthcare. Yet, a specific conceptualization of health as related to the environment has not been provided in public health and in philosophy. In medical education for instance the role of the environment appears to be often compartmentalized with respect to health, despite calls for the development of an ecological and environmental account of health (see Coope, 2021).

## Health as coping with the environment

To take the first steps towards reclaiming the role of the environment and develop a relational account of health and environment, we go back to some lesser-known official WHO documents. In the 1980s, WHO developed a major health promotion program, which was followed by an interesting revision of the idea of health. The working group on “Concepts and Principles in Health Promotion” published an official document which called for health promotion based on a socioecological approach to health (WHO, 1984). The document innovatively conceptualizes health as: “the extent to which an individual or group is able to realize aspirations and satisfy needs; and, on the other hand, to change or cope with the environment. Health is therefore seen as a resource for everyday life, not the objective of living; it is a positive concept, emphasizing social and personal resources, as well as physical capacities” (WHO, 1984, *Principles*). This definition is taken up in the Ottawa Charter (WHO, 1986), which is the result of the first conference on global health promotion organized by WHO, Health and Welfare Canada, and the Canadian Public Health Association, and it is recognised as a pivotal document for healthcare systems around the world. The charter lists “a stable ecosystem” and “sustainable resources” among the prerequisites for health. Moreover, it identifies six areas of action for health promotion, among them: to “create a supportive environment”. However, the 1984 and 1986 documents are not widely known, and most academic work and medical education textbooks tend to lean on the 1948 definition of health.^6^

Two important elements of these documents need to be put into evidence. One is that the characterization of health is ultimately related to *the capacity to cope with the environment*. The other, pragmatic aspect, is the need to *create a supportive environment* to promote health and prevent disease. However, these official documents and the limited subsequent debate fail to provide a detailed definition of these concepts. While they constitute an interesting starting point for considering the environment in relation to health, they do not provide an extensive characterization of what it means to cope with the environment and create a supportive environment.

Interestingly, the Editorial of another special issue of *The Lancet* (2009), written in relation to climate change, emphasises that the individual’s health cannot be considered as separated from the environment, as the individuals do not live in a biological vacuum. The very title of this editorial is significant as it addresses health in terms of the capability to adapt: “What is Health? The Ability to Adapt”. However, the idea of “ability to adapt” advocated in this editorial still needs an elaborated theoretical foundation. To achieve this, we need to look at biological theory. We develop this approach in the next two sections. We focus on the ideas of “cope with the environment” and “create supportive environments”, by discussing two notions, adaptation and adaptivity, in the context of the debate on health. The idea of ‘creating supportive environments’ is then further discussed through two case studies of adaptivity in Sect. 6.

## What does ‘cope with the environment’ mean? The concept of adaptation in public health

In medicine the term ‘coping’ is used with different meanings. One is individual based. It is often associated with the notions of ‘adjustment’ and ‘adapting’ in the sense of how individuals react to their own health states over time (Saolomon & Murray, 2002). Coping and adjustment refer to the evaluation of the perceived health and derive from self-report assessments. In this context, adapting and adaptation refer to change in the true health levels over time, such as variable proficiency in the use of the right hand if the left one is impaired (Salomon & Murray, 2002, p. 620).

A second meaning of ‘coping’ is still centred on the individual, but refers to the changes of the individuals in response to their environment. This meaning is closer to that employed by the 1984/1986 WHO documents to introduce the role of the environment in the characterization of health. However, this idea can be taken much further to investigate the relationship between health and environment in such a way as to situate health and go beyond an individual centred perspective.

To do so, and develop a relational account of heath that include the role of the environment, we propose to focus on the concepts of *adaptation* and *adaptivity*, as developed in two different research fields: public health and philosophy of biology, respectively. In public health, adaptation is being used in relation to climate change and the effects of ecological issues/disasters on health and well-being. Several definitions of adaptation have been provided (e.g., the one by the United Nations and the Intergovernmental Panel on Climate Change (IPCC, 2022)). Currently, one of the most comprehensive definition is provided in the context of climate change by the US Global Research Program, which defines adaptation as “the adjustment in natural or human systems to a new or changing environment that exploits beneficial opportunities or moderates negative effects” (US GCRP, 2018, p. 1487; see also Conlon & Austin, 2021).

Adaptation refers thus to a great number of strategies and interventions related to climate sensitive exposure, which practitioners and public managers can assess and use. These actions are aimed at reducing risks and the adverse effects of climate change on the health and well-being of vulnerable individuals and communities. Among the adverse effects of climate change on health we can mention heat-waves, hurricanes, vector-borne diseases, tick borne diseases etc. (see also Balbus et al., 2016). Vulnerable individuals and communities are usually considered on the basis of age, socioeconomic status, immigration status, geographical location, etc.

Adaptation is different from mitigation, even though the two terms are closely related. Mitigation is about primary prevention, such as the reduction of greenhouse gas emissions in public and private sectors and the implementation of the use of green energy (see for instance mitigation related activities in the Forth National Climate Assessment, NCA4 2018; see also Martinich et al. 2018). Adaptation concerns, instead, the secondary prevention in public health (Bierbaum et al., 2014, p. 671). In this context, adaptation implies that collective and interdisciplinary actions should not only aim to mitigate the effects of climate change (e.g. reduction of CO_2_ emissions), but should cope with and prevent climate hazards. In this way, the term ‘adaptation’ has also been adopted in the context of architecture and landscape management (Alizadeh & Hitchmough, 2019). It conveys the assumption that collective and interdisciplinary actions have to be considered not only to mitigate the effects of climate change (e.g. reduction of CO2 emissions), but to cope with and to prevent climate hazards by ameliorating buildings, public spaces and lands policies in cases of adverse natural events. This means organising, planning, forecasting interventions at different scales to reduce the vulnerability of categories and communities and to increase resilience against adverse effects of climate change.^7^

The value of adaptation in public health and in healthcare management is out of discussion. However, a limit of this approach is that the environment, especially in medicine, is often characterized as exclusively pathogenic. In the majority of cases, the environment is identified *tout court* with the negative effects of climate change or with adverse situations. In this context, it is considered a source of perturbation to which both medicine and healthcare practice must effectively respond. The problem of this approach is that, in some way, it fails to consider the preventive role of the environment in fostering health. Adaptation measures to the environment appear to be mainly focused on individuals and to be responses a posteriori (for instance after a disruptive meteorological event or after the spreading of a disease). An extensive preventive policy of promoting supporting environments is often missing in the adaptation-based strategy.

This is due at least in part to the conceptualization of the environment implied in the adaptation measures. From the theoretical and practical points of view, a ‘pathogenic approach’ is not sufficient to provide an understanding of the health outcomes of the environment. A better and more fine-grained characterization of the idea of *coping* with the environment needs to be provided. In the next section, we turn to how the notion of adaptation from public health can be complemented with the idea of adaptivity from philosophy of biology and theoretical biology, and can also provide insights into possible practical applications.

## What does ‘cope with the environment’ mean? The notion of adaptivity

Adaptivity is a concept employed in contemporary systems science, theoretical biology, and cognitive science. It is the capability of a system, such as an organism, to remain viable in its environment by regulating itself (Di Paolo, 2005), and it is usually related to physiology and behaviour.^8^ Generically speaking, it is the ability to “cope” with changes in the environment.

From the historical point of view, the roots of this notion can be traced back to two research lines at the crossroad of physiology, systems science, and cybernetics. One line starts in physiology with Claude Bernard’s (1865) notion of constancy of the internal *milieu*, later developed by Walter Cannon into the notion of *homeostasis* (1929), and it focuses on the capability of organisms to maintain some variables stable. Another influential account of adaptivity is provided by Jean Piaget (1967) who, instead, emphasises change over stability. Piaget understands adaptivity as the capability of an organism to respond to environmental perturbations by means of internal reorganisations.^9^

Theoretical biology and philosophy of biology have recently emphasised the importance of focusing on adaptivity to understand the relationship between the organism and its proximal environment (see Heylighen, 2002; Di Paolo, 2005; Barandiaran & Egbert, 2013; Bich et al., 2016). However, in most cases, adaptivity has been given a generic or descriptive characterization as a form of stability: “a system’s capacity to adjust to changes in the environment without endangering its essential organization” (Heylighen, 2002, p. 25). In the simplest and most general scenario, organisms can be considered as capable of living only within those environmental “boundary conditions” (represented as an attractor) that allow their persistence (Barandiaran & Egbert, 2013).

More recent work in philosophy of biology and theoretical biology has focused on investigating the types of mechanisms that organisms can employ to cope with variations both in their environment and in their own physiology. By pursuing this approach, Bich et al. (2016) distinguish between “stability” and “adaptive regulation”. *Stability* is characterised as a passive network property: the system simply “absorbs”, as a network, the effects of perturbations or internal variations. It does so by compensating for them through internal reciprocal adjustments between tightly coupled subsystems. As a result, the whole dynamic is maintained in the initial attractor, or it is pushed by the perturbation into a new stable attractor. *Adaptive regulation* is characterized, instead, as the active modulation of the internal dynamics and behaviour of a system in relation to variations in internal and external conditions. Such modulation is carried out by means of specialized mechanisms that evaluate perturbations and operate accordingly. The main idea is that keeping the system viable requires selecting (and modulating) the appropriate operations to perform, given specific circumstances. In the case of network stability, the organism responds passively to the environment. *Regulatory adaptivity*, or *adaptive regulation* enables, instead, the organism to actively engage with the environment through change (Bich et al., 2020).

Living systems, including human beings, are continuously interacting in changing environments, and are constantly exposed to their effects (see the notion of *exposome,* Miller, 2020). Adaptivity—as developed in these recent studies—is a relational concept that describes both the regularity and the variability of certain behaviours in changing conditions.

In sum, the notion of adaptivity and, the more specific nuance of adaptive regulation, is valuable as it provides a precise and fine-grained characterization of the interactions between organism and environment. In the section we turn to how this characterization can be fruitfully applied to the relationship between health and environment.

## The notion of adaptivity to understand the health-environment relationship: what we can learn from other organisms

Both concepts analysed above—*adaptation* and *adaptivity*—provide tools to understand the relationship between health and environment in terms of the capacity to adapt, and its implications for current scenarios.

Adaptation, as employed in public health, marks an important step in understanding the negative effects of the environment. Thus, for example, the notion of adaptation proves valuable in forecasting, encouraging, and planning the necessary measures to be taken in public health and in health policy with respect to climate change. It has been argued that the study of adaptation to climate change should become an integral part of medical education in order to develop an ecological understanding of the notion of health (Coope, 2021; Rapport et al., 2003).

Yet, as we have underlined, the notion of adaptation is usually applied to responses to ‘negative’ or ‘pathogenic’ aspects of the environment. Moreover, the idea of the creation of *supportive environments,* introduced in the 1984 WHO definition of health, and adopted in some of the most recent definitions of adaptation in public health, requires to be further developed.

The notion of adaptivity can be of valuable help in this respect. Adaptivity, as analysed in the most recent approaches in philosophy of biology, could be of interest for public health, medical education, and philosophy of medicine as it relies on a more complex and fine-grained idea of the environment which, moreover, is not merely identifiable with climate change or with a pathogenic account. As such it can be of help to understand the general importance of the environment for health.

Adaptivity allows to make a principled distinction between two ways of conceptualising a relationship between the environment and the organism, and its relation to health. The first is based on stability and the idea of returning to the initial state of the system (or one of its variables) after a perturbation (such as in the generic notion of homeostasis). The second consists in actively bringing forth adaptive changes in the system (such as in the adaptive regulation). These two approaches are not necessarily mutually exclusive: the first might be a special case of the second (Bich et al, 2016). Yet they may convey different assumptions about the system and the environment and support different strategies in relation to health.

The environment is a rich and complex concept that does not have a precise and universal characterisation.^10^ In the human case it may include built spaces, natural spaces, generic surroundings, technological contexts, etc. It is difficult to define by itself, as its definition(s) would need to include entities that interact with it and within it. Let us look, for example, at a simple dictionary definition of environment as: “the complex of physical, chemical and biotic factors that act on an organism or ecological community and ultimately determine its form and survival” (Encyclopaedia Britannica, 2020). This definition includes the entities that interact with an environment. In this case, the interaction implied by this definition is just unidirectional: from the environment to the organism or the ecological community. Accounts of the environments may change depending on whether one focuses on unidirectional or multidirectional interactions, or on a global (macroscopic), proximate (mesoscopic) or individual (microscopic) scale. This concept seems to escape a single, unified, definition and to require a multifaceted and multi-scalar analysis.

For these reasons, our purpose is not to provide a full-fledged definition of environment, but to develop an approach and a perspective from which to characterize the relationship between a system and its environment in different cases, and clarify its roles in health. The notion of adaptivity provides such an approach and it allows to reframe this concept. On the adaptivity account, the environment is characterized relationally. It does not constitute a set of independent boundary conditions affecting a system. Moreover, the interaction with the environment is not characterized in negative terms. Adaptivity entails a different approach, focused on engaging with and taking advantage of variability and change, instead of preventing it. Regulatory mechanisms do not only respond conservatively to perturbations that menace the survival of the system or destabilise some variables in the system. A system endowed with adaptive regulatory mechanisms can make decisions on the basis of what it senses in the environment (Bechtel & Bich, 2021). From this perspective, the interaction with the environment is constitutive of a biological system, which needs to manage positive and negative interactions in such a way as to maintain itself viable.

On this view the environment ceases to be just a generic source of noise or perturbations that menace the system and need to be counteracted (such as in an approach based on stability). This is important insofar as grounding the idea of *coping* with the environment in terms of disturbances, implicitly conveys a partial view of the environment as mainly pathogenic: a source of hazards that need to be blocked, counteracted, or eliminated. Such pathogenic view of the environment associated with health, tends to overlook the positive or preventive interactions aimed at fostering, rather than just re-establishing health. If we think of the current pandemic, for example, it is easy to find this negative view in expressions commonly used in the media such as the virus as “the enemy” or “we are at war with the virus” or “going back to normal”.

On the contrary, a first reframed characterization of the relationship between organism and environment which emerges from the notion of adaptivity is to see this relationship as a source of opportunities that allow a system to expand its range of viability. Some examples from a range of living organisms can be of clarification. Not only humans, but all living organisms—starting from bacteria—employ regulatory mechanisms to actively create or exploit opportunities in the environment rather than counteract its influence. For example, by means of regulatory mechanisms bacteria can follow a gradient of concentration of nutrients, synthesise different enzymes to metabolise different nutrients depending on their availability in the environment and their energy efficiency, establish themselves in given locations or to move to others, etc.

It is important to specify that the point of view of the internal physiology of the organism does not exhaustively account for the relationship with the environment. Organisms do not only regulate themselves to respond to or take advantage of their environment, but also employ regulatory mechanisms to act in and modulate the environment to promote their conditions of existence. Living organisms adaptively *cope with the environment*, and to do so they *create supportive environments*. Even bacteria already show this capability. They employ a variety of strategies to gain access to and create different biological environments (Baldari et al., 2006; Lemonnier et al., 2007). Collectively, organised in colonial systems such as biofilms, they also produce complex dynamic extracellular molecular structures, the bacterial EPS matrix (Steinberg & Kolodkin-Gal, 2015) which, among other things: (1) favour the colonisation of new environments (Lopez et al., 2009); (2) organize differentiated micro regions in space, where groups of cells or mixed-species micro consortia locally share a similar extracellular environment and work together (Flemming & Wingender, 2010); (3) realize an external digestive and signalling system (Dragoš & Kovács, 2017); (4) build channels that harness the flow of liquids and nutrients (Cairns et al., 2014).

Animals, too, exert a regulatory control upon the abiotic and biotic environment to maintain themselves viable, either by directly harnessing and modulating the external flux of matter and energy or by indirectly generating and regulating external structures in the environment such as bird nests, spider webs, beaver dams, etc. (Christensen & Bickhard, 2002; Nunes Neto et al., 2014). These environmental structures might in in turn act as regulatory mechanisms and modulate the conditions of existence and the behaviour of the organism that produces them, as well as those of other living systems.^11^

Turning to humans, the role of these mechanisms is even more evident. Let us just think about water management. The control of the availability and distribution of water is essential for the creation of supporting environments. Water is fundamental for several human activities from food to energy production, etc. Most of all, drinking water is necessary for human life, and health is deeply affected, positively and negatively, by the quality of water. Moreover, the effect of water on health can depend on the type of infrastructures available. This is coherent with a multiscale and relational approach to the role of the environment. Let us think of the recent water crisis in the city of Flint, Michigan, US (see Ruckart et al., 2019). In April 2014, for economic reasons, the source of drinking water for the city was changed from the Lake Huron to the Flint River, whose water is more corrosive. As a consequence, the water distribution pipes, made of lead, which started leaching out, rapidly provoked serious effects on the health of the inhabitants of Flint, especially children. The effects included, among others, slow brain growth and development, resulting in cognitive problems. After Government officials finally acknowledged the problem based on the concerns voiced by citizens, the source of city water was shifted back to the Huron Lake, but the corroded pipes kept leaking. The interventions carried out by health agencies, among which the Centers for Disease Control and Prevention/the Agency for Toxic Substances and Disease Registry (CDC/ATSDR), and by the *The Flint Lead-Free Initiative*, constitute an example of adaptive regulatory mechanisms, characterized by two steps: (1) the evaluation of the problem, including both the investigation of the state of the distribution infrastructure and the screening of the population; (2) the intervention on the water distribution system. Such intervention did not simply consist in going back to the previous source of water. This was not sufficient due to the damage already caused by the corrosive river water to the lead pipes, that kept leaking. It required changing the system, in this case by starting to replace the pipe network and the lead-based fixtures in thousands of homes. Such intervention was accompanied by several others focused on prevention and on providing the citizens with access to doctors and to other healthcare, education and social services.

Going back to the discussion about the 1984/1986 WHO definition of health, this situated and relational view of regulatory adaptivity may give more substance to the idea of health as “coping with the environment”. The notion of health should not only include environmental noise and potentially threatening perturbations, but also the capability to modulate the relationship with the environment to promote the conditions of existence of the system, that is, to create supportive environments.^12^ Let us consider another example: a situation such as the current pandemic, where the perturbation is the virus SARS-CoV-2 responsible for the disease COVID-19. A first important strategy of response may be the one of *blocking the perturbation* or bringing the system back to its initial state.^13^ It consists in preventing the virus from spreading in the first place. For example, efforts are made to eliminate the source of perturbation by monitoring potential communities of host organisms for potential viruses that might jump to humans (Cui et al., 2019; Ge et al., 2016). Another essential strategy, which was successful in the case of SARS in 2002, consists of establishing quarantine areas and travel bans, social distancing, and use of face masks, etc. (Abramo, 2021; Wilder-Smith et al., 2020).

However, not all perturbations, or their sources, can be blocked or reverted. Other, less obvious, adaptive strategies imply modulating the relationship with the environment at different scales to make humans and their environments better able to *manage the effects of a perturbation*. In this case the environment becomes a fundamental part of the adaptive response rather than a source of perturbation only. In the next section, we discuss two case studies of this type of adaptive strategy: community-based medicine and the bioinformed design of microbiologically healthier buildings.

## Creating supportive environments: community-centred care and bioinformed design as case studies

Coping with the environment and creating supportive environments may require strategies and mechanisms at different scales. In the 1984/1986 WHO definitions, health is characterized as “a resource for everyday life, not the objective of living” (WHO, 1984). Referring to “everyday life” implicitly includes many different scales in which the environment can be intertwined with health. It is possible to focus on the *micro* (which affects the individual organism), *meso* (which affects a group) and *macro scale* (which globally affects all individuals).^14^ In the human case, adaptivity at the micro scale may include, for example, changes in individual behaviour in the environment as well as medical treatments provided by health professionals within healthcare facilities. Adaptivity at macro scales may include policies and interventions to counter climate change, and travel restrictions in case of pandemics. In this section, we focus on the *meso* scale, where most of adaptive interaction with the environment happens and which affects groups and individuals on an everyday basis.

In order to clarify our situated and relational account of health we discuss two recent case studies. They are examples of adaptive strategies to create supportive environments that are currently being promoted or which could be better implemented in response to the COVID-19 pandemic. Together, the following two case studies show how the environment in a broad sense (natural and built), is a fundamental tool for fostering health and well-being.

The first case study focuses on community-centred care, a topic discussed in public health, medical theory and healthcare practice. Community-centred care consists in the expansion of healthcare outside hospitals towards communities. It is a complex approach, rich in applications, which has been widely studied by healthcare professionals and humanities scholars in order to improve and empower communities with respect to their health and well-being (Arxer & Murphy, 2018). Community-centred care, people-centred care, integrated care, home-based community-integrated care for targeted patients, and cultural-based care are just few recent examples of the different developments of this approach, which has been acquiring greater importance in the last decades (Fabbri et al., 2020; Skemp, 2017; Van der Vlegel-Brouwer et al., 2020).

Community-centred care has been considered both an integral part of medical practice and a possibility to act directly on territories and within communities. The underlying motivation for this approach is that that centralized health care systems (mainly hospitals) have failed to recognize the role of communities and of the individuals that compose a community situated in a specific territory (Torres et al., 2014; Valles, 2018, p. 192).

The community-centred approaches to care are not simply based in a community, but they are aimed at mobilizing assets within the communities, in order to promote equity, justice and empower people towards the control of their health and their lives (Freudenberg & Tsui, 2014). In 2020, during the COVID-19 pandemic, the World Health Organization (WHO) published a guide to help countries to foster the resilience of community-based health services, by improving home and community-based services and collaborations with local based NGOs (WHO, 2020). As it is specified in the document: “Community-based health care includes services delivered by a broadly defined community health workforce, according to their training and capacity, encompassing a range of health workers, lay and professional, formal and informal, paid and unpaid, as well as facility-based personnel who support and supervise them and provide outreach services and campaigns” (WHO, 2020, p. 4). In maintaining health service and in strengthening the COVID-19 response, the community-centred approach requires engaging with local communities, private individuals, health professionals at different levels, such as prevention, control, screening, home care and cure of patients.

During the first year of pandemic an implementation of the community-centred care principle has been advocated among health professionals. This debate shows how a practice used in medicine can be adapted to the current scenario and can constitute itself a form of adaptivity in healthcare.

The starting point is a letter published in the *NEJM Catalyst* by a group of Italian MDs and researchers (Nacoti et al., 2020) followed by a perspective paper (Nacoti et al., 2021). Both in the paper and in the letter, the Italian MDs denounce the inadequateness of patient centred care and call for its replacement with community-centred care. The authors work at the Papa Giovanni XXIII Hospital in Bergamo, a brand-new facility (with 48 intensive care unit (ICU) beds) situated in one of the epicentres of the pandemic in Italy. According to official data, from February to April 2020 more than 2000 people died of COVID-19 just in the town of Bergamo (which has a population of 122,243 inhabitants). The context described in the letter and in the paper is discouraging as denouncing the high number of deaths and the exhaustion of healthcare professionals (Nacoti et al., 2020, p. 2). This situation, shared by many health facilities in Italy and around the world from February 2020, has been widely denounced (Barello et al. 2020; Blake et al. 2020). It has also been accompanied by the recognition of the very important work of the health professionals and the attempt to plan for better management of the ICU beds (Gómez-Moreno et al., 2020; Manca et al., 2020).

Interestingly, Nacoti et al., (2020, 2021) focus not just on the numbers of beds in an ICU and the numbers of professionals available in a given situation, but, on the “where” of the caring. The paper claims that the pandemic showed that the Western health system, which has been built on the concept of patient-centred care, focused on the individual, needs to be refocused towards community-centred care.

It is known that patient-centred care (PCC) focuses on the patient’s needs, health, and well-being. Its importance has been widely recognised, for example with regards to the autonomy of the patient in medical decisions (Slater, 2006; Taylor & Taylor, 2013). PCC (and by extension family-centred care) interventions have been demonstrated as effective for the perception of the quality of care, self-care behaviours, reduction of the intensity of stress, depression, etc. (Park et al., 2018).

On our view, a community-centred approach does not exclude PCC, but calls for changes in public health along with better long-term plans. After saying that “we are learning that hospitals might be the main COVID-19 carriers, as they are rapidly populated by infected patients, facilitating transmission to uninfected patients” (Nacoti et al., 2020, p. 3, see also Nacoti et al., 2021), the authors propose a community-centred model based on home care and mobile clinics, as they would limit hospitalisation to a specific target group of patients, relieve the pressure on healthcare facilities, and be more effective in limiting the spread of contagion. The Italian experience shows an example of the need for adaptivity in healthcare management during this pandemic and the importance of taking the environment into account. It demonstrates that it is not viable to confine care within the walls of the hospitals. This has implications not only in the case of a pandemic event but for healthcare in general. COVID-19 highlighted the importance of a medicine grounded in the communities and territories. It is an opportunity to not only vindicate a form of medicine that serves to combat crises in which the environment is pathogenic (e.g. with the presence of a virus), but also to implement permanent measures that improve adaptive mechanisms as a way to develop contexts that are more supportive of human health. Adaptivity in this sense means a series of (innovative) measures to face a threat (such as the diffusion of a virus) and to monitor the health of the community in an anticipatory way. Health and the conceptualization and treatment of disease change as the result of the relationship between the community, its values, and the public/private places where healthcare and preventive medicine are based.

There are many other examples of adaptive mechanisms supporting and promoting health and well-being through interaction with the environment. The second case study focuses on the built environment and more specifically, on bioinformed building design.

The current pandemic has brought to our attention the role of pathogens in the air inside and outside of buildings.^15^ Research on the airborne spreading of viruses in indoor spaces has increased, together with a call for better design and management of built environments to prevent the spread of airborne pathogens and therefore of respiratory infections.

It has been underlined that: “For decades, the focus of architects and building engineers was on thermal comfort, odour control, perceived air quality, initial investment cost, energy use, and other performance issues, whereas infection control was neglected” (Morawska et al., 2021, p. 689). Accordingly, the monitoring, control, and maintenance of threshold value of CO_2_ inside buildings have been recommended, as well as health-based indoor quality guidelines (e.g., WHO guidelines for indoor air quality about threshold levels of benzene, carbon monoxide, etc.). Less attention has been paid to the dynamics involving viruses and bacteria in the air inside buildings, until the recent development of research on bioinformed design of microbiologically healthier buildings (Green, 2014; Horve et al., 2020; Li et al., 2021). This research investigates the possibility of controlling the interaction between bacteria inside and outside public and private buildings in order to achieve better working and living conditions.

The case of microbiologically healthier buildings is another example of an adaptive mechanism involving the environment, which could constitute a step forward in coping with COVID-19 and future epidemics and pandemic events. Research on bioinformed design is an important extension of theoretical research on the microbiome, a relevant topic in science and in philosophy of science in the last decades (O’Malley & Parke, 2020). This approach starts from acknowledging that humans, as well as the spaces they inhabit, are colonized by microorganisms: every one of us “aerosolises around 37 million bacteria per hour” (Yong, 2016, p. 251). Living human spaces are inhabited by bacteria and viruses, coming from human bodies, from those of visitors, friends, pets, from outside air, etc. This applies to homes, public buildings, schools, universities, and hospitals. It has been underlined how bacteria and viruses in the air, and spaces more generally, are not necessarily dangerous for human health. On the contrary, evidence has been provided for the case of air-conditioned hospital rooms that harmless microbes from outdoors air, plants and soil are necessary to create a healthy diversity and turnover that through competition prevents pathogenic microbes from spreading or developing resistance to antibiotics and disinfectants (Green, 2011). This topic can be traced back to the history of modern medicine, such as in the pioneering work of Florence Nightingale on ventilating hospital rooms of wounded soldiers during the Crimean War (Yong, 2016, p. 257; Allitt, 2021).

In the last year, it has been highlighted that the simple gesture of ventilation could have been an effective measure to stop the spreading of COVID-19. Unfortunately, its importance has been recognised too late by many building managers, architects, designers, and common people, together with the difference between the role of droplets and aerosols in the spreading of the COVID-19 (Polianskjy, 2021). Opening a window is just the first adaptive mechanism in creating a supportive environment which fosters health in buildings during COVID-19 times. Even though very important in preventing the spreading of airborne diseases, manual ventilation is not enough in many situations. Other adaptive measures have been considered to realize microbiologically healthier buildings, among them flexible mechanical ventilation depending on various purpose, number of occupants, and measures to save energy (Morawska et al., 2021, p. 689; see also Morawska & Cao, 2020). Furthermore, in order to “shape” the microbiological community within public and private buildings, biomedical research encourages architects and managers to learn how to maintain or even grow certain types of microbiomes, for example by rethinking cleaning practices, allowing for the presence of pets, fostering the use of daylight, using specific materials in construction—such as natural wood—providing humidity control, and implementing microbiome detection instruments (Dannemiller, 2019; Dietz et al., 2020).

All these elements taken together are adaptive measures in the built environment that do not only fight against the current virus, but also contribute to users’ health. Therefore, as a consequence of this pandemic, not only medicine but also architecture is encouraged to rethink its practice and take into account the relationship between indoor and outdoor environments and the health of occupants (microbes, animals and humans).

## Conclusion

In this paper we addressed the relationship between health and environment by integrating contributions from philosophy of medicine, environmental philosophy, and philosophy of biology and by taking into account challenges which emerged during recent years. We posit that to understand health, now and for the future, the environment has to play a pivotal role. We have discussed definitions of health provided by the WHO and argued that any approach to health and environment should develop two key ideas from the 1984/1986 definitions: “coping with the environment” and “creating supportive environments”. Even if these ideas are present in some documents and innovative medical approaches, they have still not been given enough attention. To fill this gap, we have built upon the notion of adaptation and adaptivity developed in medical theory and philosophy to provide a framework that relate the environment to health. While arguing that it is not sufficient to say that organisms “cope” with their environment more or less successfully, we developed an approach to health focused on the adaptive regulatory mechanisms that articulate the intricate connections between organisms and environment. Adaptivity, as developed in this paper, characterizes the dynamic relationship between organism and environment in terms of changes brought forth in both systems by specialized mechanisms in response to variations. This notion allows us to reflect on health in terms of how humans and environments can be made more capable to not just to react to but to *manage the effects of perturbations*.^16^ On this view, the environment is not only a source of perturbations, but most importantly it becomes a fundamental part of the adaptive change. This implies the creation of supportive environments. We have discussed two recent examples of this type of adaptive strategy which are particularly relevant in the current scenario and have implications for healthcare, architecture, and management: community-based care and the bioinformed design of microbiologically healthier buildings.

Further studies, both theoretical and practical, need to be conducted to better understand the relationship between health and “*coping*” with the environment. This paper provides a first interdisciplinary step in this direction.

## Footnotes

1 Recent works that analyse the debates and the current state of the philosophy of medicine include Broadbent, 2019; Fuller, 2017, 2018; Kingma, 2019; Saborido, 2020; Solomon et al., 2017; Stegenga, 2018.

2 See https://health.gov/healthypeople.

3 Boorse and many other naturalists (Ananth, 2008; Gammelgaard, 2000) tend to identify this idea of the normal environment with the historical context in which the design of organisms has been evolutionarily shaped. Interestingly, this approach is somehow at odds with some of the work done in physiology during the second part of the twentieth century, focused on studying the physiology of humans and other biological organisms out of laboratories in different ecological settings (desert, artic, etc.) or in variable environments (day/night conditions, variation of temperatures etc.) (see Hagen, 2021).

4 It is worth noting that normativism aligns with the aforementioned definition of the WHO Constitution as it directly links health to the subjective notion of complete physical, mental and social well-being (WHO, 1948).

5 As a consequence, it might contribute to overlooking again the wider role of the viruses in the history of the evolution and in ecology (Chadarevian & Raffaetà, 2021).

6 Significantly, in a period of increased attention given to urgent environmental issues, such as in the last few years, a search on Google Scholar shows that between 2016 and 2020 the WHO 1948 definition of health has been cited 782 times, while the 1984 definition has been cited only 48 times. See also Starfield, 2001 and Huber et al. 2011.

7 See for instance the Building Resilience Against Climate Effects (BRACE) framework which is five-steps assessment method for health administrators and health professionals to: (1) forecast climate impacts and assessing vulnerabilities; (2) project the disease burden; (3) assess public health intervention; (4) develop adaptation plans; (5) evaluate the impact and improve the qualities of activities. https://www.cdc.gov/climateandhealth/BRACE.htm; (Marinucci et al., 2014).

8 Biological *adaptivity* is different from *biological adaptation*, although the uses and meanings of these terms are often overlapping. Adaptation is a central concept in evolutionary biology, which refers to the fit between an organism and its environment caused by natural selection (Lloyd, 2021). Thus, the evolutionary concept of adaptation encompasses the different mechanisms that explain the evolution of organisms with respect to environmental pressures, which cause changes in organisms that are explained in terms of differences in fitness. The notion of *biological adaptation* is therefore mainly historical (Allen, 2002; Williams, 1966). To avoid confusion, hereinafter we will use the term *adaptivity* as the organism’s capability to cope with a changing environment, unless specified otherwise.

9 The distinction between approaches focused on stability and change is not sharp, and these two ideas often overlap. This is evident in the history of theoretical and experimental work on homeostasis in physiology and ecology, where different notions, such as heterostasis and allostasis, have been introduced to revise and further develop the idea of homeostasis to explicitly take into account the role of change (see for example, Selye, 1975; Schulkin 2004; Sterling & Eyer, 2005; Arminjon, 2016). It is beyond the scope of this paper to investigate this history, as the aim here is rather to identify the conceptual basis of these two dimensions of the notion of adaptivity and discuss their implications for an understanding of the relationship between health and environment. For an historical analysis of the development of this idea and a discussion of the debate on the relationship between stability and change in homeostatic systems, see for example Hagen (2021).

10 See for example Di Paolo (2020) and Pearce (2010) for historical work.

11 There might be connections, worth exploring in future work, between these ideas focused on adaptive mechanisms and theoretical work on niche construction and affordances (Gibson, 1979; Walsh, 2015), and on the relationship between health and affordances (Menatti & Casado, 2016).

12 This approach is supposed to complement, not replace, the physiological view of health, focused on taking care of sick patients. A good definition of health should still take into account—albeit not exclusively—what happens within living beings. For a perspective focused on health and the role of regulatory mechanisms in physiology see Saborido and Moreno (2015), Saborido et al.,(2016) and Bich et al., (2020).

13 For pioneering theoretical work on this type of strategy, from a cybernetic approach, see Ashby (1956, 1958) and Conant and Ashby (1970).

14 For reasons of space, we cannot elaborate too much on this distinction between micro, meso and macro scales. It is a distinction "of degree" which must be refined on a case-by-case basis in order to be explanatorily useful. For example, it is a distinction that makes sense in cases where a particular disease and its treatment are understood differently if one looks only at the properties of an individual's immediate environment or of a much broader environment, which may even encompass the entire world population, as in the case of a pandemic. An example is an asymptomatic carrier of a pathogen, whose potential effect vary if one considers the immediate or a broader environment.

15 See for example a recent theme issue of the journal *Interface Focus* on the ‘Airborne transmission of SARS-CoV-2’ (Tang et al., 2022); and also Morawska et al. (2021).

16 Interestingly this approach is in line with the new IPCC report issued in February 2022 (IPCC, 2022). A section of IPCC 2022 is entitled: ‘Impacts, Adaptation and Vulnerability’. The introduction of this section of the report seems to employ a more dynamical idea of adaptation than in previous reports: “Adaptation in ecological systems includes autonomous adjustments through ecological and evolutionary processes. In human systems, adaptation can be anticipatory or reactive, as well as incremental and/ or transformational. The latter changes the fundamental attributes of a social-ecological system in anticipation of climate change and its impacts” (IPCC, 2022, p. 35). Moreover, the concept of resilience, which plays an important role in adaptation measures, is considered in its broader meaning. Not just ‘bouncing back and returning to a previous state after a disturbance’ and ‘the ability to maintain essential function, identity and structure, but also the capacity for transformation” (IPCC, 2022, p. 35).
